# Supplementary material for: The genome sequence of the Yellow-barred Brindle, Acasis viretata (Hübner, 1799) (Lepidoptera: Geometridae)
Source: Wellcome Open Res. 2025 Sep 15;10:516. [Version 1] doi: 10.12688/wellcomeopenres.24888.1 (PMC12519069; doi:10.12688/wellcomeopenres.24888.1)
Supplement: Peer review history

# Reviewer report: Reviewer response for version 1

**DOI:** 10.21956/wellcomeopenres.27415.r133500

**Published:** 2025-10-24

**Authors:** Yu-Feng Huang

**Affiliations:** 1 Computer Science and Engineering, Yuan Ze University (Ringgold ID: 34895), Zhongli District, Taoyuan City, Taiwan; 2 National Chung Hsing University, Taichung, Taiwan

This manuscript presents a high-quality, chromosome-level genome assembly of *Acasis viretata*, the Yellow-barred Brindle moth. The study forms part of the Darwin Tree of Life (DToL) initiative and follows well-established protocols for DNA extraction, sequencing, assembly, and curation. The genome exhibits strong assembly metrics, including a scaffold N50 of 17.88 Mb, a BUSCO completeness score of 98%, and 99.98% assignment to 17 chromosomes (including the W and Z sex chromosomes).

 Overall, the manuscript is technically robust, clearly written, and well aligned with the goals of the Tree of Life gateway. It can be recommended for publication after addressing the following minor but important clarifications.

 1. Mitochondrial Genome Characterization

- While the mitochondrial genome (16,010 bp) has been assembled, it is only briefly described. The unit “kb” is not appropriate for this scale; it should be expressed in base pairs (bp).
- The authors should verify and explicitly state whether the mitochondrial genome is circularized and complete, as circularity is a key quality metric for mitogenome assemblies.
- It is also recommended to clarify whether the mitochondrial genome sequence was validated or polished, since linear assembly outputs can occasionally represent incomplete circular genomes.

2. Assembly Completeness and Curation

- The k-mer completeness discrepancy (primary = 78.42%, alternate = 71.98%, combined = 98.80%) warrants a short discussion. The authors should clarify whether the missing content in the primary assembly is represented in the alternate haplotype or reflects possible heterozygosity collapse.
- **Assembly curation:** Although the manuscript briefly mentions the chromosome curation process, the discussion is limited. The statement “manual corrections included 4 breaks and 9 joins” provides insufficient detail. It would be helpful to specify whether these corrections occurred at the scaffold or chromosome level and to summarize their rationale or impact on overall assembly quality.

3. Clarification in the “Nucleic Acid Extraction” Section

 In the subsection *“Nucleic acid extraction”* within the *Methods* section, the second paragraph includes the sentence:

 “For this sample, the final post-shearing DNA had a Qubit concentration of 28 ng/μL and a yield of 1 271.20 ng, with a fragment size of 16.9 kb.”

 The reported DNA yield value (“1 271.20 ng”) appears unusually formatted and may require verification. The authors should confirm whether this is a typographical error (e.g., a misplaced space) or an accurate measurement.

Are sufficient details of methods and materials provided to allow replication by others?

Yes

Is the rationale for creating the dataset(s) clearly described?

Yes

Are the datasets clearly presented in a useable and accessible format?

Yes

Are the protocols appropriate and is the work technically sound?

Yes

Reviewer Expertise:

Bioinformatics, Phylogenetics, Evolutionary

I confirm that I have read this submission and believe that I have an appropriate level of expertise to confirm that it is of an acceptable scientific standard, however I have significant reservations, as outlined above.

# Reviewer report: Reviewer response for version 1

**DOI:** 10.21956/wellcomeopenres.27415.r133495

**Published:** 2025-10-13

**Authors:** Arun Arumugaperumal

**Affiliations:** 1 Department of Biotechnology, Rajalakshmi Engineering College, Chennai, Tamil Nadu, India

The data note describes the whole genome of the Yellow-barred Brindle, *Acasis viretata*. The authors have used standard tree of life sequencing protocols to sequence and assemble the genome of this moth. The assembly size reported here is 297.68 Mb spread among 17 chromosome molecules. The mitochondrial genome was also reported which is of size 16.01 kb. Since a female specimen was used the authors have obtained the W and Z sex chromosomes also. A contigN50 value of 17 Mb indicates the good quality of the genome. BUSCO completeness of 98% indicate that the genome is a near-complete one according to existing databases. The accession id provided are active. The article can be indexed. The bioproject data has an endosymbiont genome sequence also. But it was not discussed whether it was obtained during this same sequencing procedure in the data note.

Are sufficient details of methods and materials provided to allow replication by others?

Yes

Is the rationale for creating the dataset(s) clearly described?

Yes

Are the datasets clearly presented in a useable and accessible format?

Yes

Are the protocols appropriate and is the work technically sound?

Yes

Reviewer Expertise:

Bioinformatics; Genomics

I confirm that I have read this submission and believe that I have an appropriate level of expertise to confirm that it is of an acceptable scientific standard.

# Reviewer report: Reviewer response for version 1

**DOI:** 10.21956/wellcomeopenres.27415.r133494

**Published:** 2025-09-26

**Authors:** Axel Künstner

**Affiliations:** 1 University of Lübeck, Lübeck, Germany

Overall Assessment

 The study presents a chromosome-level genome assembly for *Acasis viretata* (Yellow-barred Brindle), an ecologically important moth species widespread in Britain and Europe. The authors sequenced an individual female specimen using PacBio HiFi and Hi-C technologies, producing a high-quality genome sequence scaffolded into 17 chromosomal pseudomolecules, including sex chromosomes W and Z. This represents the first high-quality genome assembly for the genus *Acasis* and provides a valuable genomic resource for the Darwin Tree of Life project.

 Strengths

 Technical Quality: The assembly achieves excellent metrics with a scaffold N50 of 17.88 Mb, 99.98% of sequence assigned to chromosomes, and strong BUSCO completeness (98.0%). The EBP metric of 7.C.Q61 exceeds the recommended reference standard of 6.C.Q40. The combined use of PacBio HiFi and Hi-C data with appropriate coverage (64× and extensive Hi-C data) is well-suited for chromosome-level assembly.

 Methodological Rigor: The authors employed standard, well-validated bioinformatics pipelines including Hifiasm, YaHS scaffolding, and comprehensive quality assessment tools. The manual curation process using PretextView with documented corrections (4 breaks, 9 joins) demonstrates careful attention to assembly quality.

 Resource Value: This genome provides an important reference for Geometridae family genomics and supports conservation genomics for this ecologically expanding species. The identification and proper scaffolding of sex chromosomes W and Z adds particular value for lepidopteran genomics.

 Documentation: Comprehensive reporting of methods, software versions, and data availability meets reproducibility standards. All data are properly deposited with appropriate accession numbers.

 Areas for Improvement

 Mitochondrial Genome Characterization: The mitochondrial assembly (16.01 kb) is mentioned but not characterized. Even basic details about gene content, organization, or comparison to related lepidopteran species would significantly enhance the resource's utility for phylogenetic and evolutionary studies.

 Assembly Completeness Discussion: The k-mer completeness of 78.42% for the primary assembly, while the combined assemblies reach 98.80%, suggests some genomic content may be relegated to the alternate haplotype. A brief discussion of this pattern and its implications would be valuable.

Are sufficient details of methods and materials provided to allow replication by others?

Yes

Is the rationale for creating the dataset(s) clearly described?

Yes

Are the datasets clearly presented in a useable and accessible format?

Yes

Are the protocols appropriate and is the work technically sound?

Yes

Reviewer Expertise:

Genomics, Tumor biology

I confirm that I have read this submission and believe that I have an appropriate level of expertise to confirm that it is of an acceptable scientific standard.
